# GLIS3 expression in the thyroid gland in relation to TSH signaling and regulation of gene expression

**DOI:** 10.1007/s00018-024-05113-6

**Published:** 2024-01-28

**Authors:** Hong Soon Kang, Sara A. Grimm, Xiao-Hui Liao, Anton M. Jetten

**Affiliations:** 1 Cell Biology Section, Immunity, Inflammation and Disease Laboratory, Research Triangle Park, NC 27709 USA; 2 Integrative Bioinformatics, National Institute of Environmental Health Sciences, National Institutes of Health, Research Triangle Park, NC 27709 USA; 3 Department of Medicine, The University of Chicago, Chicago, IL 60637 USA

**Keywords:** GLIS3, Thyroid development, Thyroid follicular cell proliferation, Thyroid hormone biosynthesis, Hypothyroidism, TSH signaling, PKA

## Abstract

Loss of GLI-Similar 3 (GLIS3) function in mice and humans causes congenital hypothyroidism (CH). In this study, we demonstrate that GLIS3 protein is first detectable at E15.5 of murine thyroid development, a time at which GLIS3 target genes, such as *Slc5a5* (*Nis*), become expressed. This, together with observations showing that ubiquitous *Glis3*KO mice do not display major changes in prenatal thyroid gland morphology, indicated that CH in *Glis3*KO mice is due to dyshormonogenesis rather than thyroid dysgenesis. Analysis of GLIS3 in postnatal thyroid suggested a link between GLIS3 protein expression and blood TSH levels. This was supported by data showing that treatment with TSH, cAMP, or adenylyl cyclase activators or expression of constitutively active PKA enhanced GLIS3 protein stability and transcriptional activity, indicating that GLIS3 activity is regulated at least in part by TSH/TSHR-mediated activation of PKA. The TSH-dependent increase in GLIS3 transcriptional activity would be critical for the induction of GLIS3 target gene expression, including several thyroid hormone (TH) biosynthetic genes, in thyroid follicular cells of mice fed a low iodine diet (LID) when blood TSH levels are highly elevated. Like TH biosynthetic genes, the expression of cell cycle genes is suppressed in ubiquitous *Glis3*KO mice fed a LID; however, in thyroid-specific *Glis3* knockout mice, the expression of cell cycle genes was not repressed, in contrast to TH biosynthetic genes. This indicated that the inhibition of cell cycle genes in ubiquitous *Glis3*KO mice is dependent on changes in gene expression in GLIS3 target tissues other than the thyroid.

**Supplementary Information:**

The online version contains supplementary material available at 10.1007/s00018-024-05113-6.

## Introduction

CH is one of the most common neonatal endocrine disorders that has been subclassified into thyroid dysgenesis caused by abnormal thyroid development and dyshormonogenesis caused by defects in thyroid hormone (TH; T3 and T4) biosynthesis [1–6]. Abnormal thyroid gland organogenesis accounts for 80–85% of patients with primary CH. During embryonic thyroid development, foregut endoderm cells give rise to thyroid progenitors, which subsequently differentiate along the follicular cell lineage leading to the formation of the TH-producing thyroid follicles [7, 8]. Several transcription factors (TFs) that are expressed early during embryonic thyroid development, including paired box 8 (PAX8), NK2 homeobox 1 (NKX2.1; TTF1), NKX2.5, forkhead box E1 (FOXE1; TTF2), and hematopoietically expressed homeobox (HHEX), play a critical role in the regulation of thyroid gland development [7]. Loss-of-function mutations in these genes cause thyroid dysgenesis [3, 6, 9, 10]. Mutations in genes critical for TH biosynthesis, including sodium iodide symporter (*NIS*; *SLC5A5*), pendrin (*PDS*; *SLC26A4*), thyroglobulin (*TG*), thyroperoxidase (*TPO*), thyroid stimulating hormone receptor (*TSHR*), and dual oxidase 2 (*DUOX2*), have been causally linked to thyroid dyshormonogenesis [11–13].

The Krüppel-like zinc finger transcription factor, GLI-Similar 3 (GLIS3), plays a critical role in the regulation of many biological processes, including thyroid follicular cell functions, and has been implicated in several pathologies [14, 15]. Loss-of-function mutations in the *GLIS3* gene are recessive. Biallelic loss-of-function mutations in human *GLIS3* leads to a multi-organ phenotype that includes congenital hypothyroidism [16–24], while monoallelic, as well as biallelic, single nucleotide polymorphisms in *GLIS3* are associated with an increased risk of thyroid dysfunction and CH [25–32]. Ubiquitous *Glis3* knockout (*Glis3*KO) mice exhibit a very similar phenotype as human patients with GLIS3 deficiency, including the development of neonatal diabetes and CH [33, 34].

The development of CH in *Glis3*KO mice was at least in part due to transcriptional repression of several TH biosynthesis-related genes suggesting that CH in *Glis3*KO mice is related to dyshormonogenesis [15, 33]. To follow-up on this study [33], we examined whether GLIS3 also plays a role in the regulation of murine thyroid gland development and whether GLIS3 transcriptional activity is modulated by TSH signaling by analyzing the expression of GLIS3 protein during pre- and postnatal murine thyroid gland. These analyses showed that the development of CH in *Glis3*KO mice is due to dyshormonogenesis rather than thyroid dysgenesis and further indicated a connection between GLIS3 protein and the TSH–TSHR signaling pathway. It is well established that TSH binding to its receptor activates several kinases that subsequently lead to the induction of gene expression, including genes involved in TH biosynthesis and cell proliferation. We provide evidence showing that TSH and activation of protein kinase A (PKA) enhance the transcriptional activity of GLIS3. We propose that the induction of several TH biosynthesis-related genes by TSH, particularly in mice fed a LID, is in part related to the increase in GLIS3 transcriptional activity [14, 15, 35]. Although, TSH also induces the expression of cell cycle-regulatory genes, our study shows that, unlike TH biosynthesis-related genes, they are not directly regulated by GLIS3 but indirectly through changes in gene expression in other GLIS3 target tissues.

## Materials and methods

### Mice and diet

*Glis3*-EGFP mice (C57BL/6-Glis3 < tm3(Glis3-EGFP)Amj >) expressing a GLIS3-EGFP fusion protein, and the ubiquitous *Glis3*-deficient *Glis3*KO1 (B6.Glis3 < tm1AmJ >) and *Glis3*KO2 (C57BL/6-Glis3 < m3(mCherry)Amj >) mice were described previously [33, 36–38]. Both mouse strains develop neonatal diabetes and CH. Conditional *Glis3* knockout mice, *Glis3*-Pax8Cre (CKO), in which the expression of Cre causes a cell type-specific deletion of exon 5 encoding zinc finger 3 and 4 of GLIS3, were generated by crossing Glis3^fl/fl^ mice [39] with Pax8Cre mice (B6.129P2(Cg)-Pax8^tm1.1(cre)Mbu^/J; Jackson Laboratory # 028196). Mice were routinely fed an NIH-31 diet (ND; Harlan, Madison, WI). For diet study, 4-week-old WT and *Glis3*-Pax8Cre mice were fed ND or a low-iodine diet (LID; TD95125 diet, Harlan) for 6 days.

### Histology and immunohistochemistry

For histological analysis, thyroid glands from different stages of mouse embryonic development and from postnatal mice were fixed overnight in 4% paraformaldehyde, washed with PBS, and embedded in paraffin. Sections (5 μm) were then stained with hematoxylin and eosin (H&E). For immunohistochemical analysis, fixed thyroid glands were washed, transferred into 30% sucrose for 2–3 days, and then frozen in OCT compound (Tissue-Tek). Frozen Sects. (10 μm) were obtained using a cryostat (Leica Biosystems, Deer Park, IL) and subsequently immunostained with the antibodies indicated. A list of primary and secondary antibodies is shown in Supplementary Table 1. Cell membranes were stained with Alexa Fluor 647-conjugated wheat germ agglutinin (WGA) and F-actin with Alex Fluor 594-conjugated phalloidin. Nuclei were stained with DAPI Prolong Diamond (ThermoFisher). Proliferation of thyroid follicular cells was analyzed by Click-iT EdU assay kit (ThermoFisher) as described previously [33]. Fluorescence was examined with a Zeiss LSM780 confocal microscope. Metamorph Premier Offline version 7.10.3.279 (Molecular Devices LLC, Sunnyvale, CA) was used to quantify GLIS3 and PAX8 positive cells. A median filter was applied to the red and green channels and Multi Wavelength Cell Scoring was used to count the total number of red cells and double positive cells.

### Measurement of serum and tissue TSH and TH levels

Serum T3, T4, and TSH levels were measured by radioimmunoassay as described in detail previously [33, 40].

### RNA-Seq analysis

Four-week-old WT (*n* = 4) and *Glis3*-Pax8Cre (*n* = 4) mice were fed an ND or LID for 6 days before thyroid glands were collected and RNA isolated with a RNAqueous-Micro total RNA isolation kit (ThermoFisher). mRNA isolation and library generation were carried out with a TruSeq Stranded mRNA and TruSeq RNA Library preparation kits (Illumina Inc., San Diego, CA), respectively. Sequences were read by paired-end sequencing using a NextSeq500 or NovaSeq 6000 Sequencing System (Illumina). Raw reads pairs were filtered to retain only those with average base quality score > 20 for both read ends. Filtered read pairs were mapped to the mm10 reference assembly via STAR v2.5 [41] with parameters "–outSAMattrIHstart 0 –outFilterType BySJout –alignSJoverhangMin 8 –limitBAMsortRAM 55000000000 –outSAMstrandField intronMotif –outFilterIntronMotifs RemoveNoncanonical". Counts per gene were extracted via featureCounts (Subread v1.5.0-p1) [42] with parameters "-s0 -Sfr -p" for RefSeq gene models as downloaded from the UCSC Table Browser on 11-07-2017. Differential gene expression analysis was performed using DESeq2 v1.14.1 [43]. For the purposes of pathway analysis, differentially expressed genes are defined at FDR threshold 0.01 and fold change > 2 or < -2. The RNA-seq expression heatmap was generated by R package ComplexHeatmap (v2.0.0) using rlog transformed values as reported by DESeq2 (v1.14.1).

### qRT-PCR analysis

RNA from thyroid glands was extracted using RNAqueous-Micro total RNA isolation kit (ThermoFisher). RNA (1 μg) was reverse-transcribed using a high-capacity cDNA reverse transcription kit (ThermoFisher) and analyzed by qRT-PCR using the TaqMan or SYBR system. QRT-PCR reactions were carried out in triplicate using a StepOnePlus Real-time PCR system (Applied Biosystem). Primer sequences are listed in Supplementary Table 2.

### Plasmids and reagents

The expression plasmid encoding constitutively active PKA (PKA catalytic subunit Calpha) was obtained from Addgene (#15310). p3xGLISBS-Luc and p3XFlag-Glis3 were described previously [33, 44]. Forskolin, FK506, and 8-Bromo-cAMP (8-Br-cAMP) were obtained from Millipore Sigma (Burlington, MA), H89 and Gö6976 from BioVision (Waltham, MA), Trametinib from Cayman Chemical Company (Ann Arbor, MI), and ZSTK474 from Enzo Life Science (Farmingdale, NY).

### Cell culture and reporter assay

Rat thyrocyte PCCl3 cells were cultured in F12 medium supplemented with 5% FBS, 1 mIU/ml TSH, 10 µg/ml Insulin, 10 ng/ml somatostatin, 10 ng/ml 1-glycyl-histidyl-lysine, 5 µg/ml apo-transferrin, and 10 nM hydrocortisone. To examine GLIS3 transcription activity, PCCl3 cells were plated in 12-well plates at 150,000 cells/well and cultured for 72 h to reach 80% confluent. Cells were grown for an additional 72 h in the absence of TSH and subsequently co-transfected with pCMV-βGal (control), p3XFlag-Glis3, and the luciferase reporter p3xGLISBS-Luc using Lipofectamine 2000 (ThermoFisher). After 24 h, cells were treated with TSH or with the compound indicated and assayed 24 h later for luciferase and β-galactosidase activity using a Luciferase assay system (Promega) and Luminescent β-gal detection kit (Takara), respectively. Luciferase activity was normalized to β-gal activity. Assays were performed in triplicate.

### Western blot analysis

PCCl3-pIND20-Glis3 cells, expressing a doxycycline (Dox)-inducible GLIS3, tagged with Flag and HA at its N- and C-terminus, respectively, were generated by infecting cells with pIND20-Flag-Glis3-HA lentivirus as described [45]. To evaluate the expression of GLIS3, PCCl3-pIND20-Glis3 cells were seeded in six-well-plate (300,000 cells/well) and cultured for 72 h, then treated with 100 ng/ml Dox for 96 h in the absence of TSH. After 24 h treatment with TSH, GLIS3-HA protein was examined by Western blot analysis and confocal microscopy. To examine degradation by proteasomes and GLIS3 protein stability, cells treated with Dox for 96 h in the absence of TSH, were subsequently treated with the proteasome inhibitor MG132 (0.5 μM) for 5 h or with the protein synthesis inhibitor cycloheximide (10 μg/ml) and TSH for the times indicated. Nuclear extracts were prepared for Western blot analysis. Briefly, cells were washed twice with cold PBS, then scraped and collected in 1.5 ml tubes. After centrifugation at 13,000 rpm for 1 min, cells were resuspended in 500 μl lysis buffer (0.5% NP-40, 100 mM Tris pH 8.0) and incubated on ice for 5 min. After a 5 min centrifugation at 13,000 rpm, nuclear pellets were resuspended in 50 μl extraction buffer (20 mM Hepes pH 8.0, 400 mM NaCl, 1 mM EDTA, 1 mM EGTA, 1 mM DTT) containing 1X Halt protease and phosphatase inhibitor cocktail (ThermoFisher), and then incubated on ice for 20 min. After centrifugation for 20 min at 13,000 rpm, supernatant was analyzed for GLIS3-HA protein by Western blot analysis with an HA antibody. Histone H3 (H3) was used as internal control of nuclear protein. Ribosomal protein S6 (RPS6), pRPS6, and NIS expression in whole cell lysates from thyroid glands of mice fed an ND or LID was examined by Western blot analysis. β-actin was used as internal control.

### Statistical analysis

*P* values were calculated by one-way ANOVA.

## Results

### Expression of GLIS3 during thyroid folliculogenesis

**Fig. 1:**
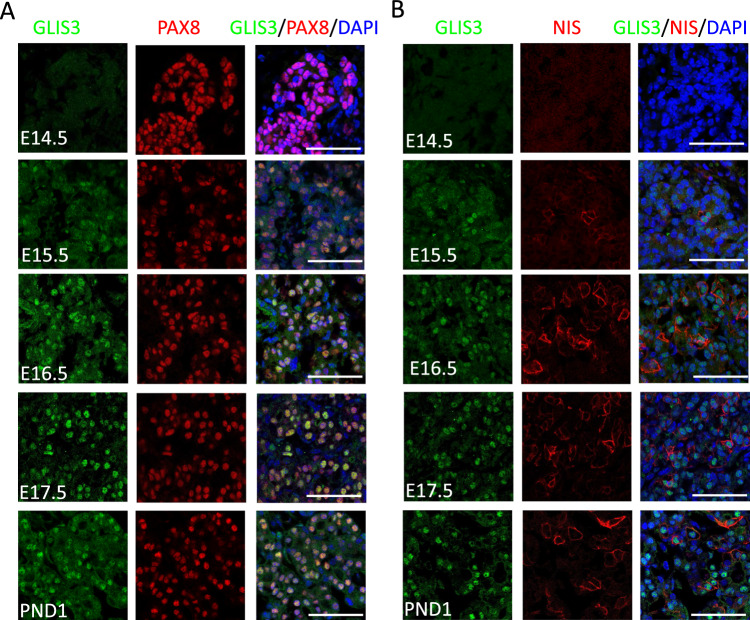
GLIS3 protein is first detectable during E15.5 of mouse embryonic thyroid gland development. **A**, **B** GLIS3 expression was examined in thyroid glands from *Glis3*-EGFP mice at E15.5, E16.5, E17.5 and PND1 by immunofluorescent staining using antibodies for GFP (GLIS3, green) and PAX8 (red) (**A**), and for GFP (GLIS3, green) and NIS (red) (**B**). Nuclei were stained with DAPI. Scale bars: 50 μm

Previously, we reported that GLIS3 protein is expressed in postnatal murine thyroid follicular cells and that it functions as a critical transcriptional regulator of several genes required for thyroid hormone biosynthesis [33]; however, whether GLIS3 plays a role in the regulation of mouse thyroid organogenesis, has not been clearly established. To investigate this further, we monitored the expression of GLIS3 protein in *Glis3*-EGFP mice at different stages of embryonic development in comparison to PAX8, which is critical for early thyroid organogenesis, and NIS, a marker of differentiated thyroid follicular cells [4, 7, 46]. In contrast to PAX8, which is already expressed at embryonic day 13.5 (E13.5), GLIS3 protein was undetectable in the thyroid gland at E13.5 and E14.5 (Fig. 1A, B, and Supplementary Fig. 1). Weak expression of GLIS3 protein was first observed at E15.5 in the nucleus of PAX8^+^ cells (Fig. 1A) and the intensity of GLIS3 immunostaining was significantly increased at E16.5. The appearance of GLIS3 protein expression at E15.5–16.5 coincided with that of the follicular cell marker NIS, which is directly regulated by GLIS3 (Fig. 1B).

**Fig. 2:**
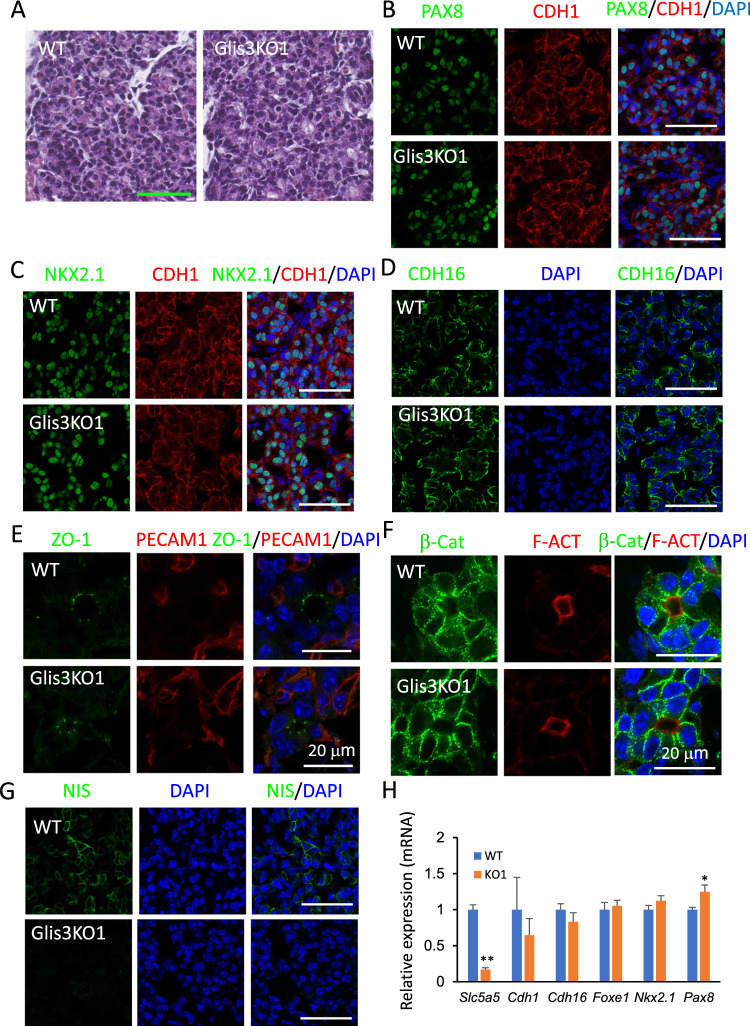
Loss of GLIS3 function does not cause major changes in mouse thyroid gland development. **A** H&E-stained sections of thyroid glands from E17.5 WT and ubiquitous *Glis3*KO1 mouse embryos. **B**–**G** Representative images of sections of thyroid glands from E17.5 WT and *Glis3*KO1 embryos immunostained with antibodies against PAX8 and CDH1 (**B**), NKX2.1 and CDH1 (**C**), CDH16 (**D**), ZO-1 and PECAM1 (**E**), β-Catenin and F-actin (**F**), and NIS (**G**). PECAM1 was used to stain endothelial cells. Nuclei were stained with DAPI. All scale bars are 50 μm, except for 20 μm in **E** and **F**. (**H**) The expression of several polarity and thyroid gland transcription factor genes was examined in thyroid gland of WT and Glis3KO1 from E18.5 embryos by qRT-PCR. WT *n* = 3, Glis3KO1 *n* = 4. *p < 0.05, **p < 0.01

To determine whether loss of GLIS3 function had any major effect on thyroid gland morphology and folliculogenesis, thyroid glands from *Glis3*KO1, in which the coding sequence of the 5th zinc finger of the DNA-binding domain was deleted [36], and *Glis3*KO2, in which the mCherry coding sequence with stop codon was inserted into exon 3 causing termination of GLIS3 translation before the DNA binding domain [33, 37], were examined by (immuno)histochemistry. Both GLIS3-deficient mouse strains develop CH. H&E histochemical staining of sections of E17.5 WT and *Glis3*KO1 thyroid glands revealed no significant differences in thyroid gland morphology (Fig. 2A) and showed a comparable pattern of PAX8 and NKX2.1 immunostaining (Fig. 2B, C). Similar observations were made with thyroid glands from postnatal day 3 (PND3) *Glis3*KO2 mice (Supplementary Fig. 2A–C).

Immunostaining further demonstrated no differences in the basolateral localization of the planar cell polarity markers, CDH1 and CDH16, and the localization of ZO-1 to apical tight junctions (Fig. 2B–E, Supplementary Fig. 2B-E) [47–49] and no change was observed in the expression of *Cdh1* and *Cdh16* mRNA (Fig. 2H). In addition, the staining patterns of β-Catenin and F-actin, which are localized to the basolateral and apical membrane, respectively, were also not altered in *Glis3*KO1 thyroid gland (Fig. 2F). These data indicate that loss of GLIS3 function did not significantly alter thyroid follicular cell polarity. Together, these observations suggest that GLIS3 does not play a major role in mouse embryonic thyroid morphogenesis. This conclusion is consistent with data showing that the expression of transcription factors that are critical for early embryonic thyroid development, were not significantly altered (*Foxe1* and *Nkx2.*1) in thyroid glands from E18.5 *Glis3*KO1, while *Pax8* expression was somewhat increased (25%) (Fig. 2H). In contrast, *Slc5a5* expression was decreased by 83% in thyroid follicular cells in E18.5 *Glis3*KO1 embryos (Fig. 2H). This was accompanied with reduced expression of NIS protein in E18.5 *Glis3*KO1 embryos (Fig. 2G) as well as in PND3 *Glis3*KO2 mice (Supplementary Fig. 2F). The observed synchronized induction of GLIS3 and NIS protein expression during embryonic development, the suppression of *Slc5a5* expression in GLIS3-deficient thyroids, and the observation that GLIS3 is expressed at E15.5, much later than PAX8 and NKX2.1 [4, 8, 50], are consistent with the hypothesis that in GLIS3-deficient mice the development of congenital hypothyroidism is due to dyshormonogenesis rather than thyroid dysgenesis [33].

### Correlation between GLIS3 protein expression and TSH levels

**Fig. 3:**
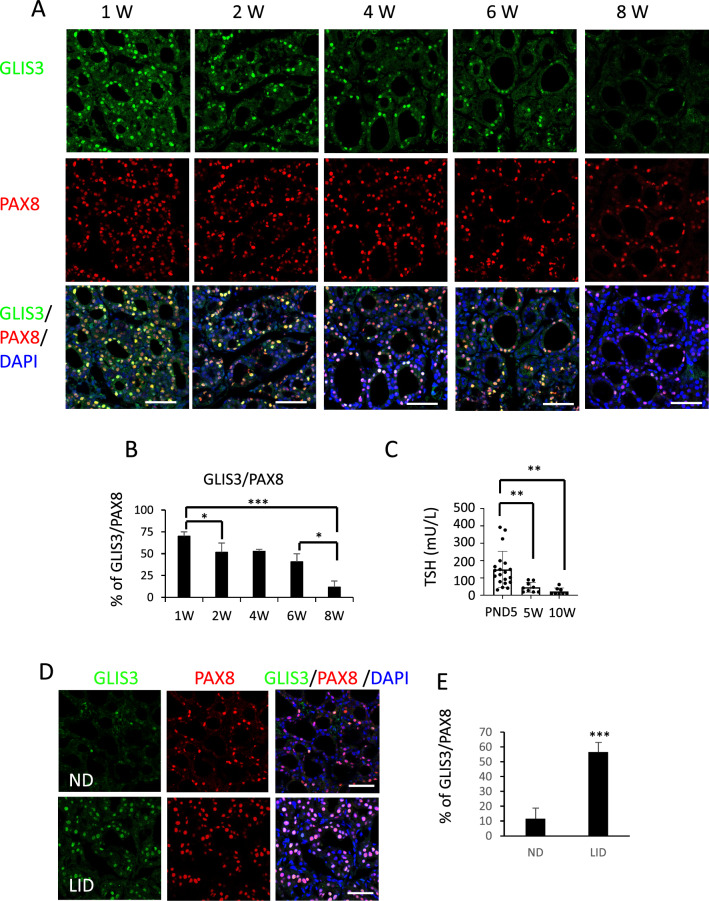
GLIS3 protein expression in thyroid follicular cells correlated with serum TSH levels. **A, B** GLIS3 protein expression is greatly decreased during early postnatal development. Expression of endogenous GLIS3 and PAX8 protein was examined in thyroid glands from 1-, 2-, 4-, 6-, and 8-week-old *Glis3*-EGFP mice by immunofluorescence staining with GFP (green) and PAX8 (red) antibodies. Nuclei were stained with DAPI (**A**). The percentage of PAX8^+^ cells that were GLIS3^+^ (from **A**) was calculated and plotted (**B**). *n* ≥ 3 for each group. Scale bars: 50 μm.** C** Serum TSH levels in WT mice were examined at PND5, 5- and 10-weeks. **p < 0.01.** D**, **E** GLIS3 protein expression in thyroid follicular cells is increased in mice fed a LID (high serum TSH). 8-week-old *Glis3*-EGFP mice were fed an ND and LID for 2 weeks before the expression of GLIS3 and PAX8 was examined in thyroid glands by immunofluorescence staining (**D**). The percentage of PAX8^+^ cells (from **D**) that are GLIS3^+^ was calculated and plotted (**E**). *n* ≥ 3 for each group. Scale bars: 50 μm

Analysis of GLIS3 protein expression during the first 2 months of postnatal thyroid development showed that GLIS3 is localized to the nucleus of thyroid follicular cells at all ages examined (Figs. 1A and 3A) consistent with previous observations [33]. However, both the intensity of GLIS3 protein staining in follicular cells and the percentage of PAX8^+^GLIS3^+^ cells in *Glis3*-EGFP mice gradually decreased over this period (Fig. 3A, B). This paralleled the steady decline in postnatal blood TSH levels (Fig. 3C). This raised the question whether there is a connection between the regulation of GLIS3 protein levels and TSH signaling. The relationship between GLIS3 protein and TSH levels was strengthened by observations showing that GLIS3 staining and the percentage of PAX8^+^GLIS3^+^ cells were greatly increased in *Glis3*-EGFP mice fed a LID, a condition in which blood TSH level is greatly elevated (Fig. 3D, E). These changes in GLIS3 protein did not show a strong correlation with alterations in *Glis3* mRNA expression (Supplementary Fig. 3) suggesting that the higher levels of GLIS3 protein expression under conditions of elevated TSH, might be due to an increase in protein stability or rate of translation rather than increased transcription.

### Link between PKA and GLIS3 stability and transcriptional activity

**Fig. 4:**
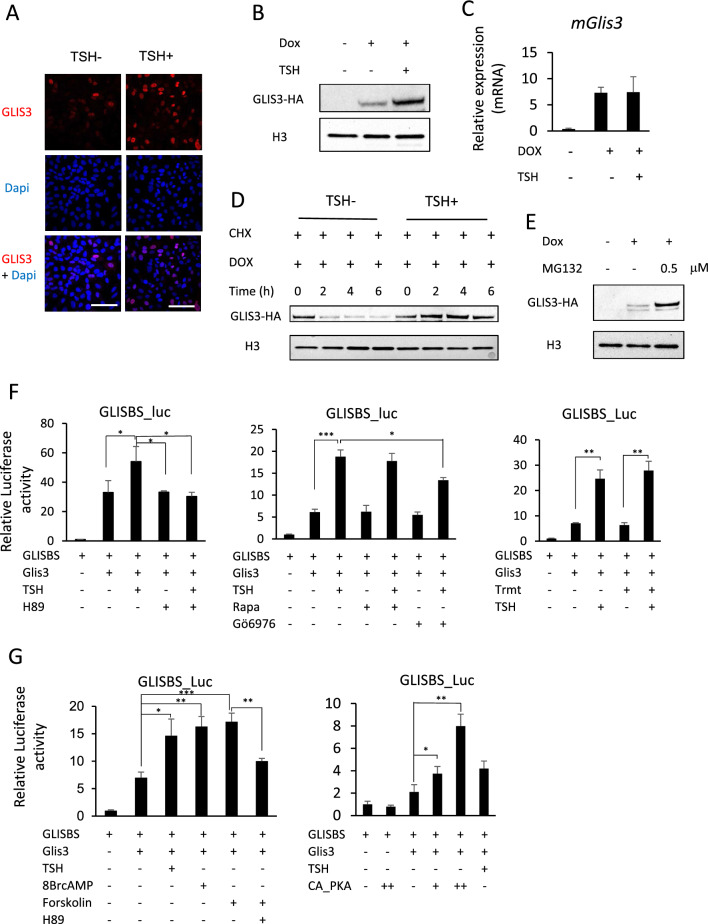
TSH and PKA activation enhances GLIS3 protein stability and transcription activity. **A**–**C** TSH enhanced GLIS3-HA protein expression in Dox-treated PCCl3-pIND20-Glis3 cells without increasing *Glis3* mRNA expression. PCCl3-pIND20-Glis3 cells were treated with Dox in the absence of TSH, then treated with or without TSH for 24 h as described in Materials and methods. GLIS3-HA protein was examined by confocal microscopy (**A**, Scale bars: 50 μm) and Western blot analysis (**B**), and *Glis3*-HA mRNA by qRT-PCR analysis (**C**). **D** PCCl3-pIND20-Glis3 cells were treated with Dox for 96 h in the absence of TSH to induce GLIS3 and subsequently with 10 μg/ml cycloheximide (CHX, to block protein synthesis) in the presence or absence of TSH. Cells were harvested at the times indicated, nuclear extracts prepared, and GLIS3-HA levels examined by Western blot analysis. **E** GLIS3 degradation by proteasomes is stabilized by MG132. PCCl3-pIND20-Glis3 cells were treated with Dox for 96 h in the absence of TSH and subsequently with or without 0.5 μM of MG132 for 5 h before cells were harvested, and nuclear extracts examined for GLIS3-HA expression by Western blot analysis. H3 was used as an internal nuclear protein marker. **F** GLISBS-dependent transcriptional activation of the Luc reporter by GLIS3 was examined in PCCl3 cells as described in Materials and Methods. Addition of TSH stimulated GLIS3-mediated transcriptional activation. This increase was inhibited by the PKA inhibitor H89, whereas treatment with the mTOR inhibitor rapamycin (Rapa), the PKC inhibitor Gö6976, or the ERK inhibitor Trametinib (Trmt) had little or slight effect on the TSH-stimulated transcriptional activation by GLIS3.** G** Addition of 8-Br-cAMP or the adenylyl cyclase activator forskolin enhanced transcriptional activation by GLIS3. The increase by forskolin was inhibited by the PKA inhibitor H89. Expression of constitutively active PKA (CA_PKA) enhanced GLISBS-dependent transcriptional activation of the Luc reporter by GLIS3. Cells were transfected with either 0.1 ( +) or 0.2 μg (+ +) PKA plasmid DNA/well. *p < 0.05, **p < 0.01, ***p < 0.001

To obtain further support for the hypothesis that GLIS3 protein expression is regulated by TSH signaling, we examined the effect of TSH on GLIS3 protein and *Glis3* mRNA expression in Dox-treated PCCl3-pIND20-Glis3 cells by immunofluorescence staining, Western blot and qRT-PCR analyses. These analyses showed that addition of TSH significantly increased GLIS3 protein expression in Dox-treated PCCl3-pIND20-Glis3 cells without changing *Glis3* mRNA expression (Fig. 4A–C). This raised the possibility that TSH might enhance GLIS3 expression by increasing GLIS3 protein stability. To examine the effect of TSH on GLIS3 protein stability, we analyzed GLIS3 protein levels in Dox-treated PCCl3-pIND20-Glis3 cells that were subsequently treated with cycloheximide in the presence or absence of TSH. Western blot analysis indicated that addition of TSH increased GLIS3 protein stability (Fig. 4D). We further showed that proteolytic degradation of GLIS3 is inhibited by the proteasome inhibitor MG132 (Fig. 4E). These data support the concept that TSH enhance GLIS3 protein stability.

Next, we investigated whether TSH also affected GLIS3-mediated transcriptional activity. The results in Fig. 4F show that addition of TSH increased GLIS3-mediated transcriptional activation of a GLISBS-dependent luciferase reporter in rat thyrocyte PCCl3 cells by 60–300% depending on the experiment; however, in the absence of exogenous GLIS3, TSH did not increase GLISBS-dependent activation (Supplementary Fig. 4). It is well established that interaction of TSH with TSHR induces the activation of several protein kinases, including PKA, phospholipase C (PLC), PI3K, mTOR, ERK, and Ca^++^-mediated signaling [7, 51–54]. To examine whether any of these downstream kinase pathways are involved in the regulation of GLIS3 activity, we analyzed the effect of several kinase inhibitors on GLIS3-mediated transcriptional activation. Addition of the PKA inhibitor H89 almost totally abolished the TSH-induced increase in GLIS3-mediated transactivation (Fig. 4F). Rapamycin, ZSTK474, and trametinib, which inhibit the mTOR, PI3K, and ERK, respectively, had little effect, while the PKC inhibitor Gö6976 reduced transactivation by about 20% (Fig. 4F and Supplementary Fig. 4). A role for PKA activation in the regulation of GLIS3-mediated transcriptional activation by TSH was supported by data showing that co-expression of a constitutively active form of PKA increased GLIS3 activity by 76% and 280% when, respectively, 0.1 and 0.2 µg PKA plasmid DNA/well was transfected into cells. Treatment with 8-Br-cAMP or the adenylyl cyclase agonist, forskolin, enhanced GLIS3-dependent transcriptional activation by 133% and 144%, respectively; this increase was significantly inhibited by H89 (Fig. 4G). Together, these results are consistent with the concept that activation of PKA is part of the mechanism by which by TSH enhances GLIS3 protein stability and transcriptional activity.

### Analysis of thyroid follicular cell proliferation in thyroid-selective *Glis3*-Pax8Cre mice

**Fig. 5:**
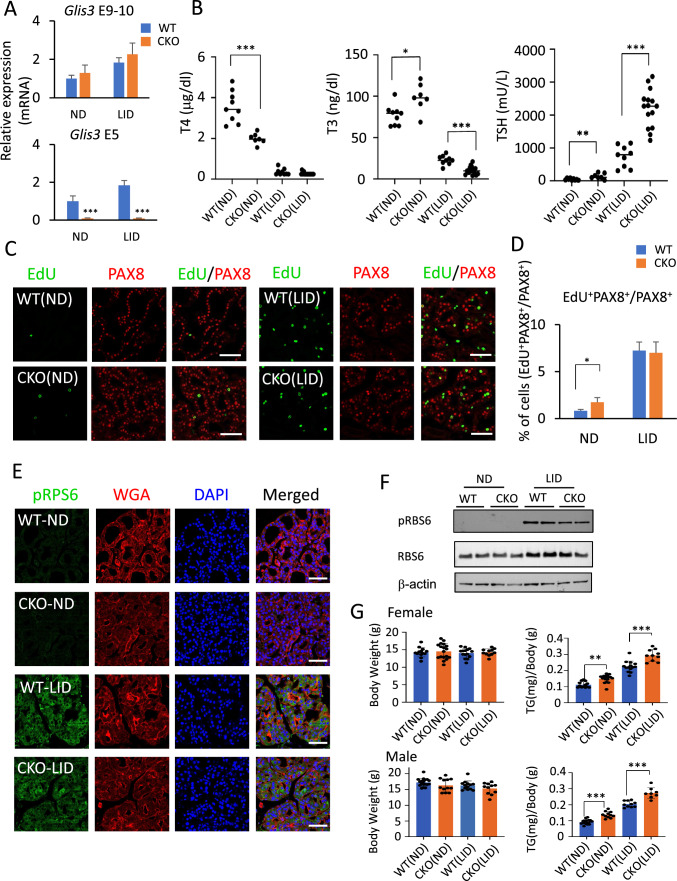
Loss of GLIS3 function does not suppress thyroid follicular cell proliferation in thyroid-selective *Glis3*-Pax8Cre(LID) mice. **A** QRT-PCR analysis of *Glis3* mRNA expression confirming the specific deletion of exon 5 (E5) in the thyroid gland of *Glis3*-Pax8Cre mice (CKO). Exon 9–10 (E9−10), a non-deleted region of *Glis3*, served as control. *n* ≥ 4 for each group. **B** Serum levels of T3, T4, and TSH in 5-week-old WT(ND), *Glis3*-Pax8Cre(ND) (referred to as CKO(ND)), WT(LID), and *Glis3*-Pax8Cre(LID) (referred to as CKO(LID)). Each dot represents one mouse. *p < 0.05, **p < 0.01, ***p < 0.001**. C** Cell proliferation in thyroid glands from WT and *Glis3*-Pax8Cre mice fed a ND or LID was analyzed by EdU incorporation (green) as described in Material and Methods. Thyroid follicular cells were stained with a PAX8 antibody (red). In contrast to ubiquitous Glis3-knockout mice [33], thyroid follicular cell proliferation was not suppressed in *Glis3*-Pax8Cre(LID) mice. Scale bars: 50 μm. **D** The percentages of the number of PAX8^+^ cells that stained EdU^+^ were calculated and plotted. *n* = 3 for WT(ND), CKO(ND), CKO(LID), and *n* = 2 for WT(LID). **E** Activation of the mTOR pathway, as indicated by pRPS6 immunofluorescent staining, was not suppressed in thyroids from *Glis3*-Pax8Cre(LID) mice. pRBS6 (green), WGA (red), DAPI (blue). Scale bars: 50 μm. ** *p* < 0.01, **p < 0.01, ***p < 0.001. F. The expression of pRBS6, RBS6 was examined in thyroid gland from WT and *Glis3*-Pax8Cre mice fed a ND or LID by Western blot analysis. β-actin was used as internal control. **G** The relative weight of thyroid glands (TG) in male and female WT(LID) and *Glis3*-Pax8Cre(LID) were increased compared to mice fed a ND, while total body weights were not changed. Each dot represents one mouse

It is well established that in addition to increasing the expression of several TH biosynthetic genes, elevated TSH levels in mice fed a LID greatly induce the expression of cell cycle genes and increase thyroid follicular cell proliferation [55, 56]. Although this increase in cell proliferation and cell cycle gene expression is suppressed in ubiquitous *Glis3*KO mice [33], very few cell cycle genes were found to be directly regulated by GLIS3 [57]. This raised the question whether the repression of cell cycle genes is caused by changes in gene expression in other GLIS3 target tissues. Because insulin-like growth factors (IGFs) and insulin play a critical role in the positive regulation of thyroid follicular cell proliferation [52, 58–62], the development of severe hypoinsulinemia in ubiquitous *Glis3*KO mice might suppress cell proliferation [15, 38, 39]. To investigate whether hypoinsulinemia contributed to the inhibition of thyroid follicular cell proliferation in ubiquitous *Glis3*KO thyroid phenotype, we analyzed the thyroid gland phenotype in thyroid-selective *Glis3* knockout mice, *Glis3*-Pax8Cre (referred as conditional knockout or CKO in the Figures), in which Pax8Cre efficiently (> 90%) deleted exon 5 in *Glis3* in the thyroid gland, but not in the pancreas (Fig. 5A and Supplementary Fig. 5A). We demonstrated that in contrast to ubiquitous *Glis3*KO mice, pancreatic insulin expression and non-fasting blood glucose levels were not changed in *Glis3*-Pax8Cre mice (Supplementary Fig. 5B, C) confirming that these mice did not develop hypoinsulinemia/hyperglycemia [63]. However, serum T4 levels were still significantly decreased (44%) in *Glis3*-Pax8Cre(ND) mice, while those of TSH and T3 were increased by 300% and 28%, respectively (Fig. 5B). Serum T4 and T3 levels were greatly decreased in both WT(LID) and *Glis3*-Pax8Cre(LID) mice fed a LID, whereas TSH was greatly increased but to a significantly greater extent in *Glis3*-Pax8Cre(LID) mice (Fig. 5B). The inconsistent change in T3 levels between *Glis3*KO(ND) and *Glis3*-Pax8Cre(ND) mice might be related to altered expression of *Dio1*, *2* or *3* in ubiquitous *Glis3*KO mice in tissues other than the thyroid gland. Consistent with this is that little change in serum T3 level was also observed in *Slc5a5* knockout mice fed a ND [64].

However, in contrast to ubiquitous *Glis3*KO2(LID) mice, the percentage of EdU^+^PAX8^+^ cells in *Glis3-*Pax8Cre(LID) mice was increased compared to *Glis3-*Pax8Cre(ND) mice and was similar to that in WT(LID) mice (Fig. 5C, D). These observations indicated that thyroid follicular cell proliferation was not repressed in *Glis3-*Pax8Cre(LID) mice. This was supported by data showing that mTOR activation, a major signaling pathway driving TSH-induced proliferation of thyroid follicular cells, was increased to a similar extent in *Glis3*-Pax8Cre(LID) and WT(LID) thyroid glands as indicated by pRSP6 immunofluorescence staining and Western blot analysis (Fig. 5E, F). The increase in thyroid follicular cell proliferation correlated with the development of thyroid gland hypertrophy in both male and female *Glis3*-Pax8Cre(LID) mice (Fig. 5G). In fact, in both male and female mice, thyroid hypertrophy was more pronounced in *Glis3*-Pax8Cre(LID) than in WT(LID) mice as indicated by the larger increase in thyroid weight (Fig. 5G). No significant difference in total body weights was observed between *Glis3*-Pax8Cre and WT mice (Fig. 5G). Together, these results suggested that GLIS3 does not play a direct role in the regulation of TSH-stimulated thyroid follicular cell proliferation and that the repression of cell proliferation in ubiquitous *Glis3*KO(LID) mice appears to be related to changes in other cell types that indirectly affect thyroid follicular cell proliferation.

### Gene expression analysis in *Glis3*-Pax8Cre thyroid gland

**Fig. 6:**
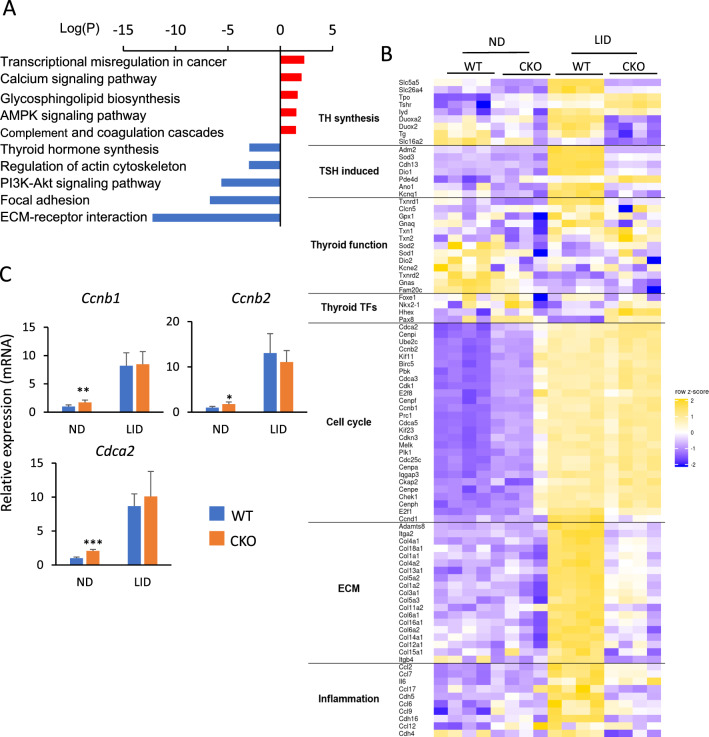
Transcriptome analysis of thyroid glands from female WT(LID) and thyroid-selective *Glis3*-Pax8Cre(LID) mice. **A** KEGG pathway analysis of genes up-regulated (red, fold change > 2; FDR < 0.01) and down-regulated (blue) in *Glis3*-Pax8Cre(LID) thyroid glands compared to those of WT(LID). **B** The relative expression of genes associated with TH synthesis, TSH induction, thyroid function, thyroid TFs, cell cycle, ECM, inflammation from RNA-Seq was visualized by heatmap. *n* = 4 for each group. **C** QRT-PCR analysis of several cell cycle-related genes. The expression of cell cycle-related genes was not repressed in *Glis3*-Pax8Cre(LID) consistent with data in Fig. 3. *n* ≥ 4 for each group. *p < 0.05, **p < 0.01, ***p < 0.001. In contrast to ubiquitous *Glis3*-knockout mice [33], cell proliferation-related genes were not suppressed in *Glis3*-Pax8Cre(LID) mice

To determine to what extent differences in the thyroid phenotype between ubiquitous *Glis3* knockout mice and thyroid-selective *Glis3*-Pax8Cre knockout mice fed a LID correlated to changes in gene expression, we performed RNA-Seq analysis (Supplementary Table 3). The transcriptome and qRT-PCR analyses were carried out with thyroid glands from *Glis3*-Pax8Cre female mice. Changes in gene expression in thyroids of female mice were like those in male *Glis3*-Pax8Cre mice and did not reveal gender differences (Supplementary Fig. 6). Analysis of up-regulated genes identified transcriptional-misregulation-in-cancer, calcium and AMPK signaling among the top pathways (Fig. 6A and Supplementary Table 4). KEGG and heatmap analysis of genes down-regulated in *Glis3*-Pax8Cre(LID) thyroids identified ECM-receptor interaction, focal adhesion, and thyroid hormone synthesis among the top pathways (Fig. 6A, B), but no cell proliferation-regulatory pathways, in contrast to ubiquitous *Glis3*KO(LID) mice (Fig. 6B) [33]. The latter was supported by qRT-PCR analysis showing that the expression of cell cycle regulatory genes, including *Ccnb1*, *Ccnb2*, and *Cdca2*, was not suppressed in *Glis3*-Pax8Cre(LID) thyroid glands (Fig. 6C) consistent with our data showing little difference in the percentage of PAX8^+^EdU^+^ cells between *Glis3*-Pax8Cre(LID) and WT(LID) thyroids (Fig. 5D). These data support the concept that the inhibition of thyroid follicular cell proliferation in ubiquitous *Glis3*KO mice appears to be related to changes in gene expression in other cell types that subsequently affect the proliferation of these cells.

**Fig. 7:**
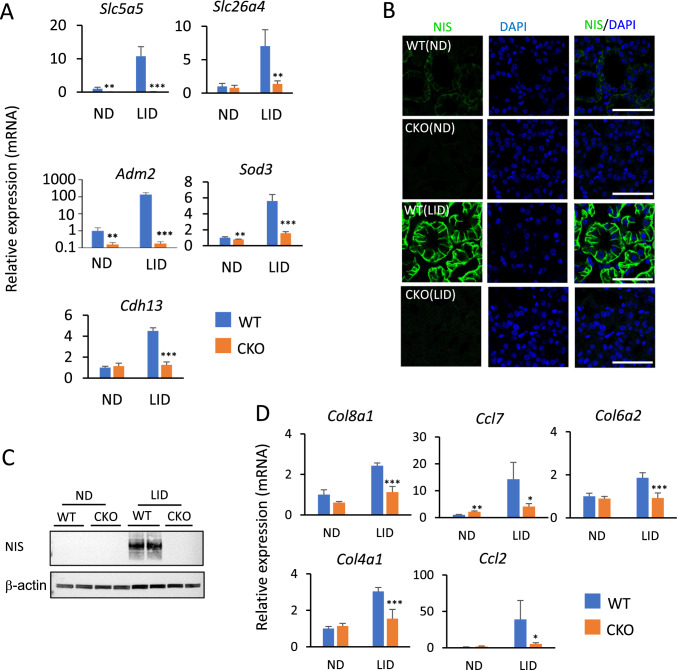
The expression of TH biosynthetic genes, ECM- and inflammation-related genes was suppressed in thyroid glands from thyroid-selective *Glis3*-Pax8Cre(LID) mice. (**A**) Comparison of the expression of several TSH-inducible genes in thyroid glands from WT(ND), WT(LID), *Glis3*-Pax8Cre(ND), and *Glis3*-Pax8Cre(LID) mice by QRT-PCR analysis. *n* ≥ 4 for each group. (**B)** The expression of SLC5A5 (NIS, green) was examined in thyroid glands from WT(ND), WT(LID), *Glis3*-Pax8Cre(ND), and *Glis3*-Pax8Cre(LID) mice by immunofluorescence staining. DAPI (blue). Scale bars: 50 μm. (**C**) The expression of NIS was examined by Western blot analysis. β-actin was used as internal control. (**D**) QRT-PCR analysis of the expression of several collagen and inflammatory genes in thyroid glands from WT(ND), WT(LID), *Glis3*-Pax8Cre(ND), and *Glis3*-Pax8Cre(LID) mice. *n* ≥ 4 for each group. *p < 0.05, **p < 0.01, ***p < 0.001

**Fig. 8:**
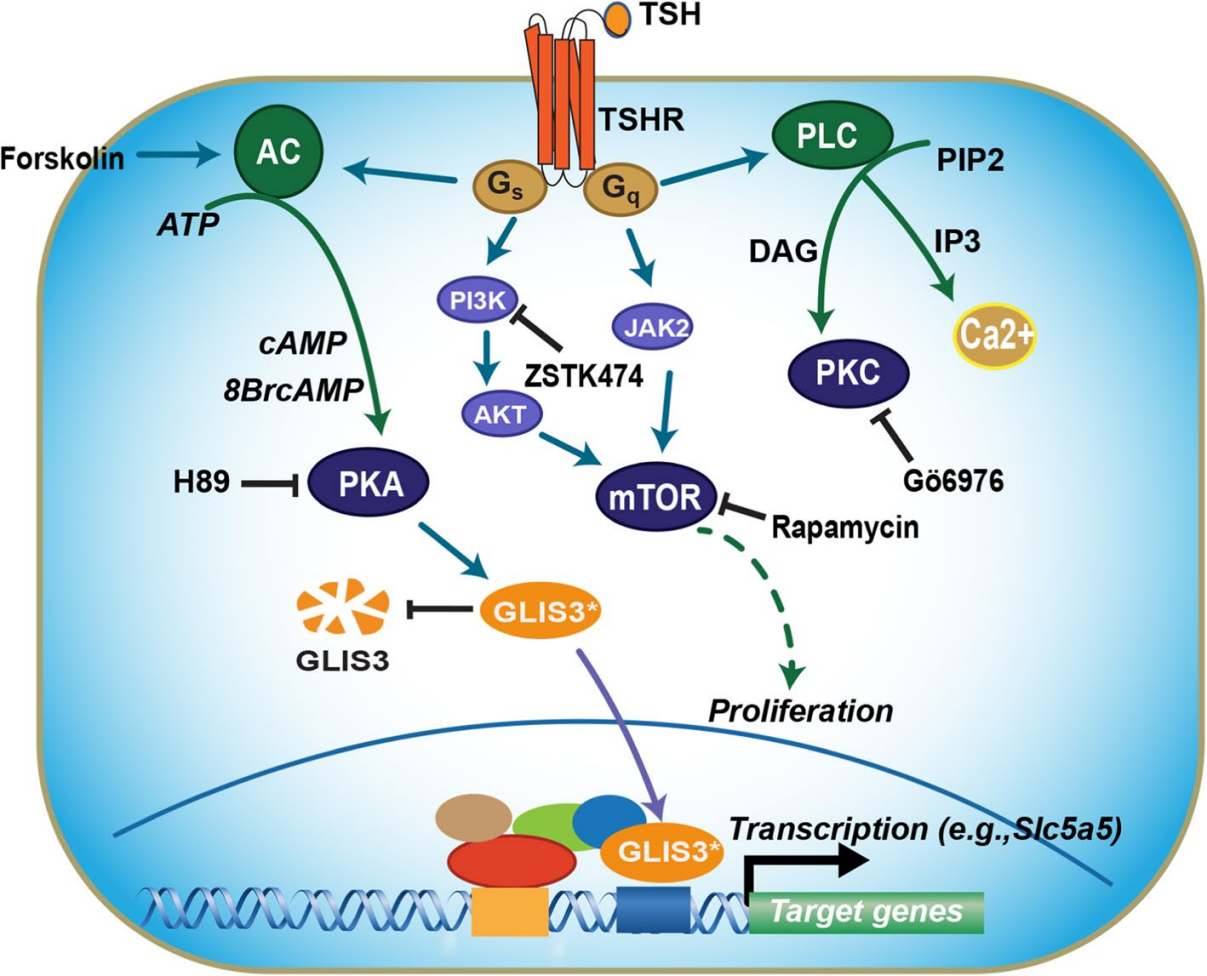
Schematic showing a model of the mechanistic connection between TSH signaling, PKA, and regulation of GLIS3 transcriptional activity. Binding of TSH to TSHR leads to activation of several protein kinase pathways. Activation of PKA by TSH, the PKA activator 8-Br-cAMP, the adenylyl cyclase activator forskolin, and expression of constitutively active PKA increases GLIS3 transcriptional activity resulting in the induction of several GLIS3 target genes, including several TH-biosynthetic genes. The PKA antagonist H89 suppressed the TSH-mediated increase, whereas inhibition of PI3K, mTOR, PKC, or ERK pathways by, respectively, ZSTK474, rapamycin, Gö6976, or trametinib, had little effect. The stimulation in GLIS3 transcriptional activity by the TSH-TSHR-PKA pathway appears to be at least in part due to increased GLIS3 protein stability. This mechanism of regulation provides an explanation for the dramatic increase in GLIS3 protein expression and the subsequent induction of GLIS3 target genes, including several thyroid hormone biosynthetic genes, in thyroid follicular cells of mice fed a LID. In contrast to TH-biosynthetic genes, cell cycle genes and cell proliferation are not directly regulated by GLIS3

Analysis of the expression of several TH biosynthetic and other TSH-induced genes, including *Slc5a5*, *Slc26a4*, *Adm2, Sod3*, *Cdh13*, *Duox2*, and *Duoxa2,* which are greatly increased in WT(LID) compared to WT(ND), were significantly repressed in thyroids from *Glis3*-Pax8Cre(LID) mice (Figs. 6B, and 7A), consistent with our previous study of ubiquitous *Glis3* knockout mice [33]. NIS protein expression was dramatically increased in WT(LID), but not in *Glis3*-Pax8Cre(LID) thyroids by immunofluorescence staining and Western blot analysis (Fig. 7B, C). Similarly, the increase in the expression of several ECM-related and inflammatory genes, including *Col18a1*, *Col6a2*, *Col4a1, Ccl7, Ccl2,* and *Itga2,* observed in WT(LID) thyroids was significantly suppressed in *Glis3*-Pax8Cre(LID) thyroid glands (Figs. 6B and 7D). Analysis of thyroid TF expression showed that *Pax8* expression was increased in both *Glis3*-Pax8Cre(ND) and *Glis3*-Pax8Cre(LID) mice compared to that of WT(ND) and WT(LID), whereas *Nkx2.1* and *Foxe1* RNA expression was not changed (Supplementary Fig. 7). This is consistent with the conclusion that the suppression of gene expression in *Glis3*-Pax8Cre(LID) thyroid is independent of changes in expression of these thyroid TFs. Together, these observations indicate that in contrast to TH biosynthetic genes, which transcription is directly regulated by GLIS3, GLIS3 does not play a major role in the direct transcriptional regulation of cell proliferation-regulatory genes in thyroid follicular cells in mice fed a LID. These findings are consistent with the concept that TSH regulates proliferation and TH biosynthesis in thyroid follicular cells through activation of different signaling pathways (Fig. 8) [7, 52, 62, 65].

## Discussion

Loss of GLIS3 function in both humans and mice causes CH [15, 16, 18, 33]. However, whether this is related to thyroid dysgenesis or dyshormonogenesis has not been clearly established. To determine whether GLIS3 plays a major role in thyroid morphogenesis, the expression of GLIS3 protein was analyzed at different stages of mouse thyroid development. This analysis demonstrated that GLIS3 protein was first detectable in thyroid follicular cells at E15.5, a time during which thyroid follicles are being formed (Fig. 1 and Supplementary Fig. 1) [4, 8]. We further show that thyroid gland morphology and the formation of thyroid follicles are not significantly altered in E17.5 and neonatal *Glis3*KO mice (Fig. 2A, Supplementary Fig. 2). These data indicate that GLIS3 is not required for early thyroid development in mice and that the development of CH in *Glis*3KO mice is due to dyshormonogenesis rather than thyroid dysgenesis. This contrasts the role of *glis3* in zebrafish, in which *glis3* has been shown to play an important role in thyroid development [66]. Moreover, unlike *glis3* knockdown in zebrafish, the expression of NKX2.1 and PAX8 was not impaired in thyroid gland in *Glis3*KO mice (Fig. 2B, C). Whether the development of CH in human patients with GLIS*3* deficiency is related to thyroid dysgenesis or dyshormonogenesis has been inconclusive and shown to vary among patients [16, 18, 20–22, 24, 25, 29, 67, 68]. This variability might be attributed to the highly oligogenic nature of CH [27, 29, 32].

Our study further shows that the onset of GLIS3 expression is distinct from that of the thyroid TFs, PAX8, NKX2.1, FOXE1, and HHEX, which are expressed much earlier than GLIS3 during embryonic thyroid development [4, 7, 8, 50, 69–71]. These TFs are critical for early thyroid gland development and their loss of function causes thyroid dysgenesis [3, 4, 6, 9, 10, 12, 72]. In addition to their developmental function, several of these TFs also play a role in the regulation of several thyroid follicular cell functions, including TH biosynthesis. This is supported by our recent study showing that GLIS3 regulates the transcription of several TH biosynthesis-related genes in coordination with PAX8 and NKX2.1 [57]. This study also demonstrated that PAX8 and NKX2.1 are bound to regulatory regions of the *Glis3* gene, which would be consistent with the concept that these factors have a role in the transcriptional activation of *Glis3* during thyroid development and act upstream of GLIS3 [33, 57]. Our analysis of GLIS3 protein during thyroid development, further indicated that the time of GLIS3 expression during E15.5 and 16.5 parallels that of NIS protein (Figs. 1B and 2G). This is in accordance with our data showing that *Slc5a5* is a major GLIS3 target gene and that GLIS3 is required for NIS expression [33, 57]. NIS repression in *Glis3*-deficient thyroids may play a major role in the impairment in iodide transport and the development of CH in *Glis3*KO mice [33] like CH development in NIS knockout mice [64]. Together, the timing of GLIS3 expression during thyroid development and its role in directly regulating TH biosynthesis are consistent with our conclusion that CH in *Glis3*KO mice is due to dyshormonogenesis rather than thyroid dysgenesis.

It is well established that TSH not only regulates TH biosynthesis but also thyroid follicular cell proliferation [11, 52, 65]. We previously demonstrated that under LID conditions, when TSH levels are high, not only the activation of thyroid hormone biosynthetic genes was suppressed in ubiquitous GLIS3-deficient mice, but also cell cycle gene expression, thyroid follicular cell proliferation, and the activation of the mTOR pathway [15, 33]. We also reported that, in addition to congenital hypothyroidism, these mice develop neonatal diabetes and hypoinsulinemia [39, 63]. IGF-1 and insulin, together with TSH, have been reported to play a critical role in the regulation of thyroid follicular cell gene expression and proliferation [52, 58–61, 65, 73] raising the possibility that hypoinsulinemia in ubiquitous *Glis3*-deficient mice might influence the function and gene expression in thyroid follicular cells. Analysis of thyroid-selective *Glis3*-Pax8Cre(LID) mice, which do not develop hyperinsulinemia, showed that in contrast to ubiquitous *Glis3*-KO(LID) mice, thyroid follicular cell proliferation, expression of cell cycle genes, including *Ccnb1*, *Ccnb2*, and *Cdca2,* and activation of the mTOR, a pathway that promotes cell proliferation, were not repressed in *Glis3*-Pax8Cre(LID) mice but induced to a similar degree as in WT(LID) mice (Figs. 5C– F and 6C). These data indicate that GLIS3 does not play a major role in the regulation of thyroid follicular cell proliferation and suggest that the suppression of thyroid follicular cell proliferation in ubiquitous *Glis3*-deficient mice does not involve direct transcriptional regulation of cell cycle-related genes by GLIS3, but is related to abnormalities in other tissues, such as hypoinsulinemia that indirectly affect thyroid follicular cell proliferation. This conclusion is consistent with our cistrome analysis showing that GLIS3 binds to very few cell cycle-related genes [57].

In contrast to cell proliferation-regulatory genes, the expression of several TH-inducible genes, including *Slc5a5, Slc26a4*, *Adm2, Sod3*, and *Cdh13,* remain dramatically suppressed in thyroid-selective *Glis3*-Pax8Cre(LID) thyroid as we observed in ubiquitous GLIS3-deficient mice [33], consistent with the conclusion that in thyroid follicular cells their transcription is directly regulated by GLIS3 [57]. Although, IGF-1/insulin through activation of PI3K has been reported to repress TSH-induced expression of *Slc5a5* [74, 75], since GLIS3 is required for its transcription, its repression in *Glis3*-Pax8Cre(LID) and ubiquitous Glis3-KO(LID) thyroids is independent of IGF1/insulin levels [33]. These findings are consistent with reports showing that the regulation of thyroid follicular cell proliferation and TH biosynthesis by TSH involve different mechanisms [7, 67, 76].

It was interesting to note that during postnatal development, GLIS3 protein expression steadily decreased together with blood TSH levels (Fig. 3A, C). Most strikingly, GLIS3 expression was greatly increased in mice fed a LID (Fig. 3D), a condition when TSH levels are highly elevated and TSH/TSHR-dependent genes are highly induced. This is also the condition when the largest repression of GLIS3 target genes was observed in thyroids from ubiquitous *Glis3*KO mice as well as thyroid-selective *Glis3*-Pax8Cre mice (Fig. 6B[33]. Together, these observations suggested a possible link between the TSH/TSHR signaling pathway, the regulation of GLIS3 protein, and the transcriptional activation of GLIS3 target genes. This was supported by data showing that TSH enhanced GLIS3-mediated transcriptional activation and GLIS3 protein expression in PCCl3 cells (Fig. 4A, B, and F). This induction was not due to a change in the level of *Glis3* mRNA expression (Fig. 4C) suggesting that it might be due to increased GLIS3 protein stability or rate of translation. We provided evidence indicating that the stimulation of GLIS3 transcriptional activity is at least in part due to increased GLIS3 protein stability (Fig. 4B, D). GLIS3 protein stability and GLIS3-dependent transcriptional activation of target genes might be controlled by posttranslational modification(s) of GLIS3 that are mediated by TSH/TSHR-induced activation of (a) downstream kinase pathway(s) [11, 52, 65, 76]. Study of the effect of several kinase inhibitors on TSH-induced stimulation of GLIS3 transcriptional activity revealed that the PKA antagonist H89 suppressed this increase, but that inhibition of several other kinase pathways (ERK, PKC, AKT, mTOR) had relatively little effect (Fig. 4F and Supplementary Fig. 4). A role for PKA in mediating the effect of TSH was supported by data showing that the PKA agonist, 8-Br-cAMP, and the adenylyl cyclase agonist, forskolin, similarly enhanced GLIS3-mediated transcriptional activation and that this stimulation was inhibited by H89. A role for PKA was further strengthened by observations demonstrating that expression of a constitutively active PKA stimulated GLIS3-mediated transcriptional activation (Fig. 4G). It is interesting to note that previous studies found a relationship also between PKA and its regulation of PAX8 and NKX2.1 transcriptional activity [77, 78]. Mass spectrometric analysis showed that GLIS3 is phosphorylated at multiple sites (unpublished data) and further studies are needed to identify the amino acid(s) within GLIS3 that are phosphorylated by PKA and critical for regulating GLIS3 protein stability and activity. Together, our study reveals a link between TSH signaling and its regulation of GLIS3 protein activity and transcriptional activation GLIS3 target genes, including several TH biosynthesis-related genes. We further provide evidence for a role of the PKA signaling pathway in mediating the transcriptional regulation of several TSH-inducible genes by GLIS3 in thyroid follicular cells (Fig. 8). Greater insights into the upstream pathways that regulate GLIS3 expression and/or its transcription activity might provide new therapeutic approaches in the management of hypo- and hyperthyroidism.

### Supplementary Information

Below is the link to the electronic supplementary material.

Supplementary file1 (PPTX 13068 KB)

Supplementary file2 (XLSX 11 KB)

Supplementary file3 (XLSX 11 KB)

Supplementary file4 (XLSX 5167 KB)

Supplementary file5 (XLSX 195 KB)

## Data Availability

RNA-Seq data were deposited in the NCBI Gene Expression Omnibus (GEO) database https://www.ncbi.nlm.nih.gov/geo/ under accession GSE207775.

## Abbreviations

GLIS3: Gli-similar 3
CH: Congenital hypothyroidism
TH: Thyroid hormone
GLISBS: GLIS-binding site
TF: Transcriptional factor
TSH: Thyroid stimulating hormone
TSHR: Thyroid stimulating hormone receptor
ECM: Extracellular matrix
NDH: Neonatal diabetes and congenital hypothyroidism
PND: Postnatal day
KO: Knockout
CKO: Conditional knockout
PKA: Protein kinase A
ND: Normal diet
LID: Low iodine diet
NIS: Sodium-iodide symporter
DUOX2: Dual oxidase 2
FOXE1: Forkhead box E1
HHEX: Hematopoietically expressed homeobox
NKX2.1: NK2 homeobox 1
PAX8: Paired box 8
PDS: Pendrin
TPO: Thyroid peroxidase
TG: Thyroglobulin
cAMP: Cyclic AMP
ChIP: Chromatin immunoprecipitation
EGFP: Enhanced green fluorescent protein
