# Supplementary material for: GLIS3 expression in the thyroid gland in relation to TSH signaling and regulation of gene expression
Source: Cell Mol Life Sci. 2024 Jan 28;81(1):65. doi: 10.1007/s00018-024-05113-6 (PMC10822819; doi:10.1007/s00018-024-05113-6)
Supplement: Supplementary file 1 — Supplementary file1 (PPTX 13068 KB) [file 18_2024_5113_MOESM1_ESM.pptx]

## Slide 1
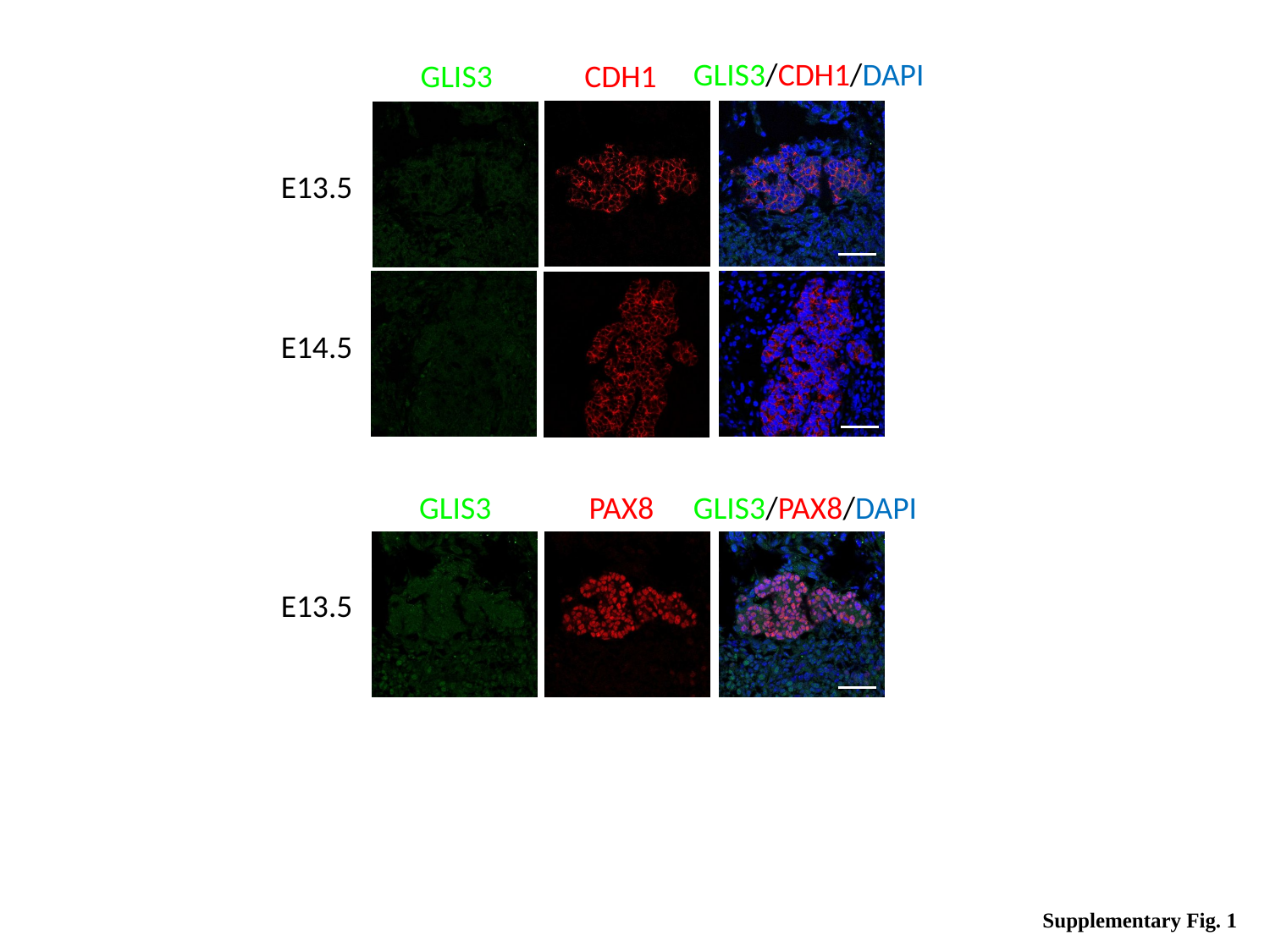

GLIS3/CDH1/DAPI
GLIS3
CDH1
E13.5
E14.5
GLIS3
PAX8
GLIS3/PAX8/DAPI
E13.5
Supplementary Fig. 1

## Slide 2
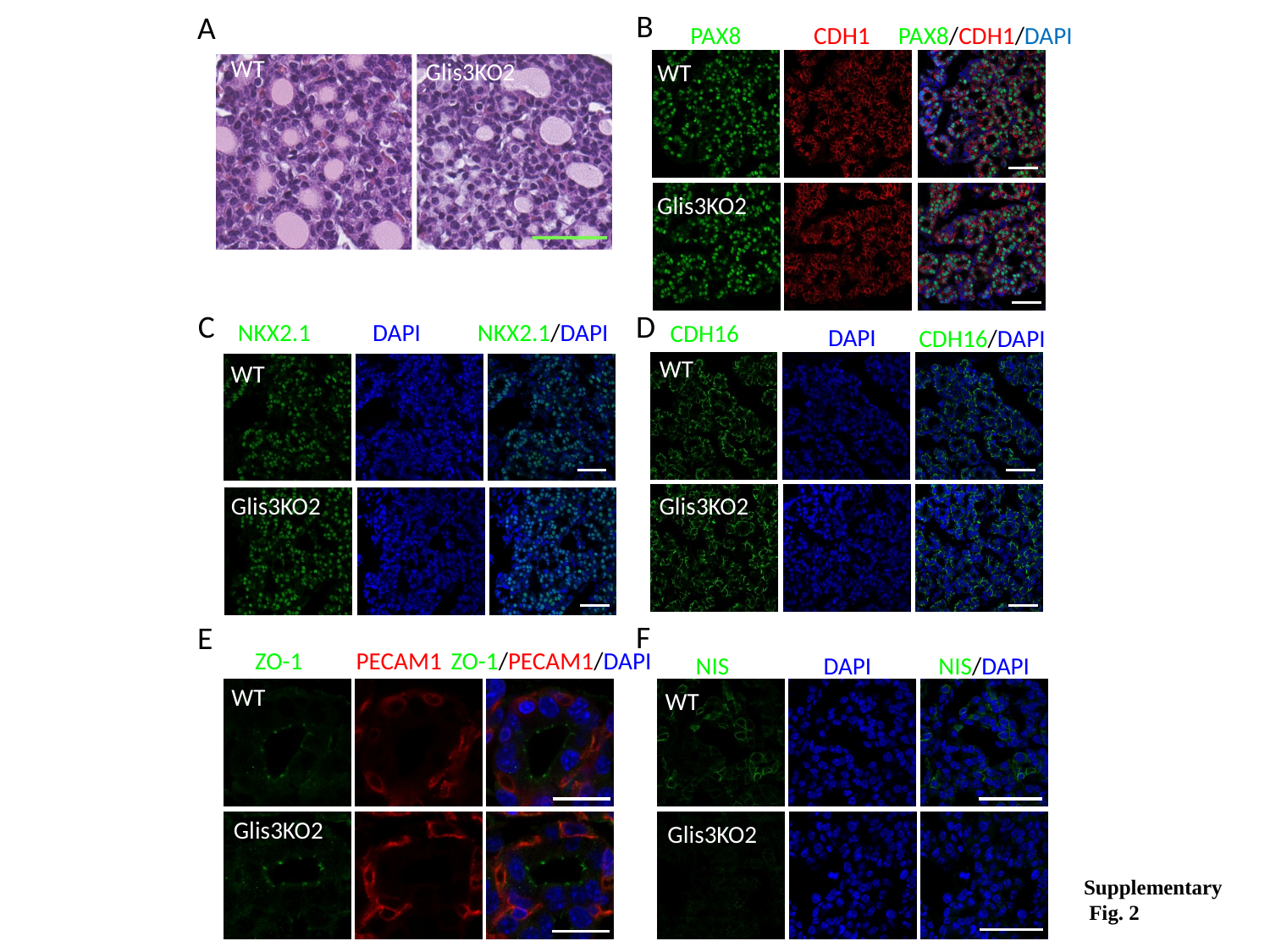

B
A
PAX8
CDH1
PAX8/CDH1/DAPI
WT
Glis3KO2
WT
Glis3KO2
C
D
NKX2.1
DAPI
NKX2.1/DAPI
CDH16
DAPI
CDH16/DAPI
Glis3KO2
WT
WT
Glis3KO2
F
E
ZO-1
PECAM1
ZO-1/PECAM1/DAPI
NIS
DAPI
NIS/DAPI
WT
WT
Glis3KO2
Glis3KO2
Supplementary
 Fig. 2

## Slide 3
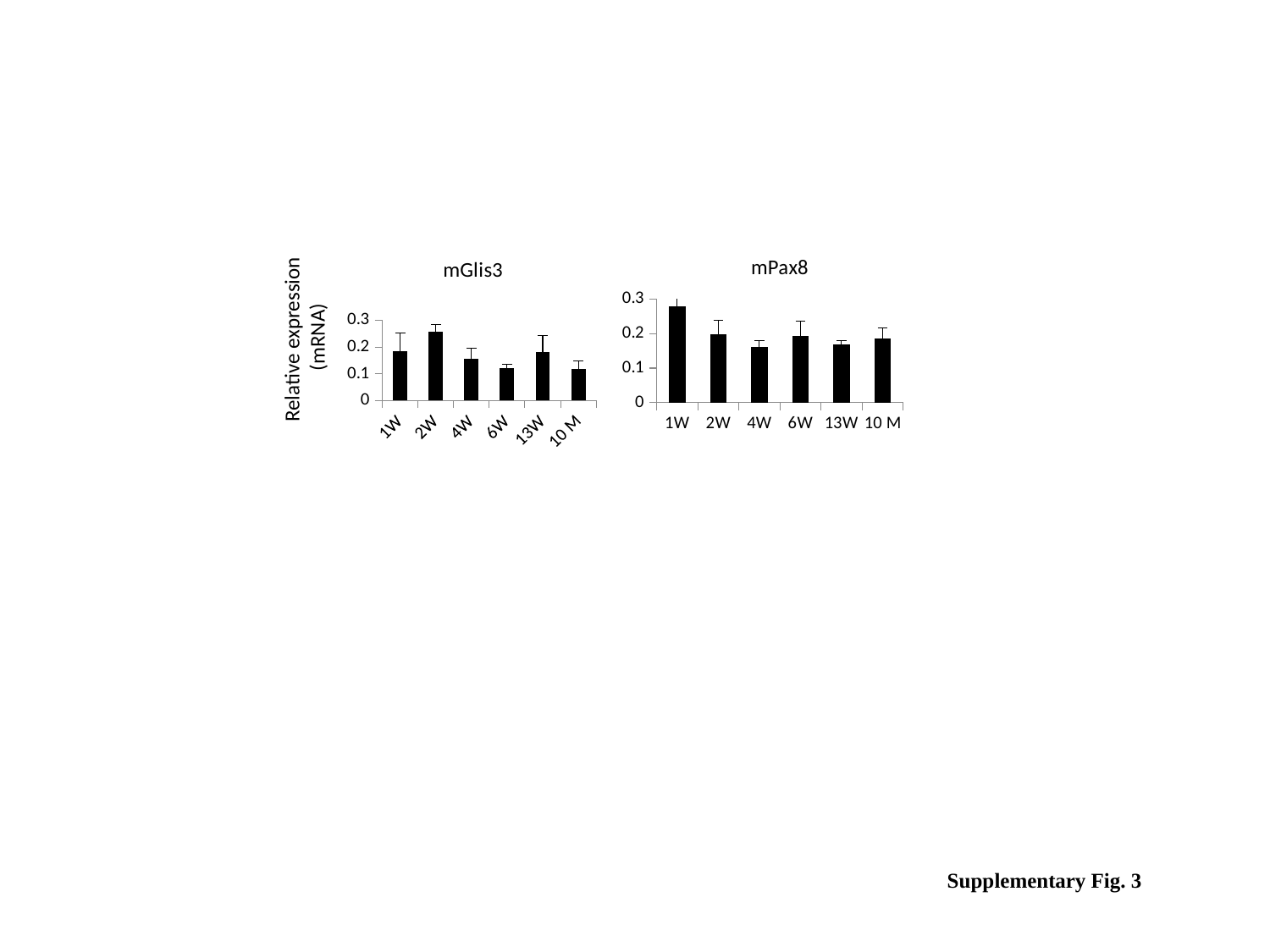

### Chart: mPax8
| Category | mGlis3 |
|---|---|
| 1W | 0.27952210051425 |
| 2W | 0.197991909254738 |
| 4W | 0.161455429032021 |
| 6W | 0.192502374352224 |
| 13W | 0.168550801565028 |
| 10 M | 0.184886025136788 |
### Chart:
| Category | mGlis3 |
|---|---|
| 1W | 0.184521525203296 |
| 2W | 0.256050973870578 |
| 4W | 0.155943681962152 |
| 6W | 0.122199417683571 |
| 13W | 0.18097030498563 |
| 10 M | 0.117429790120643 |Relative expression
(mRNA)
Supplementary Fig. 3

## Slide 4
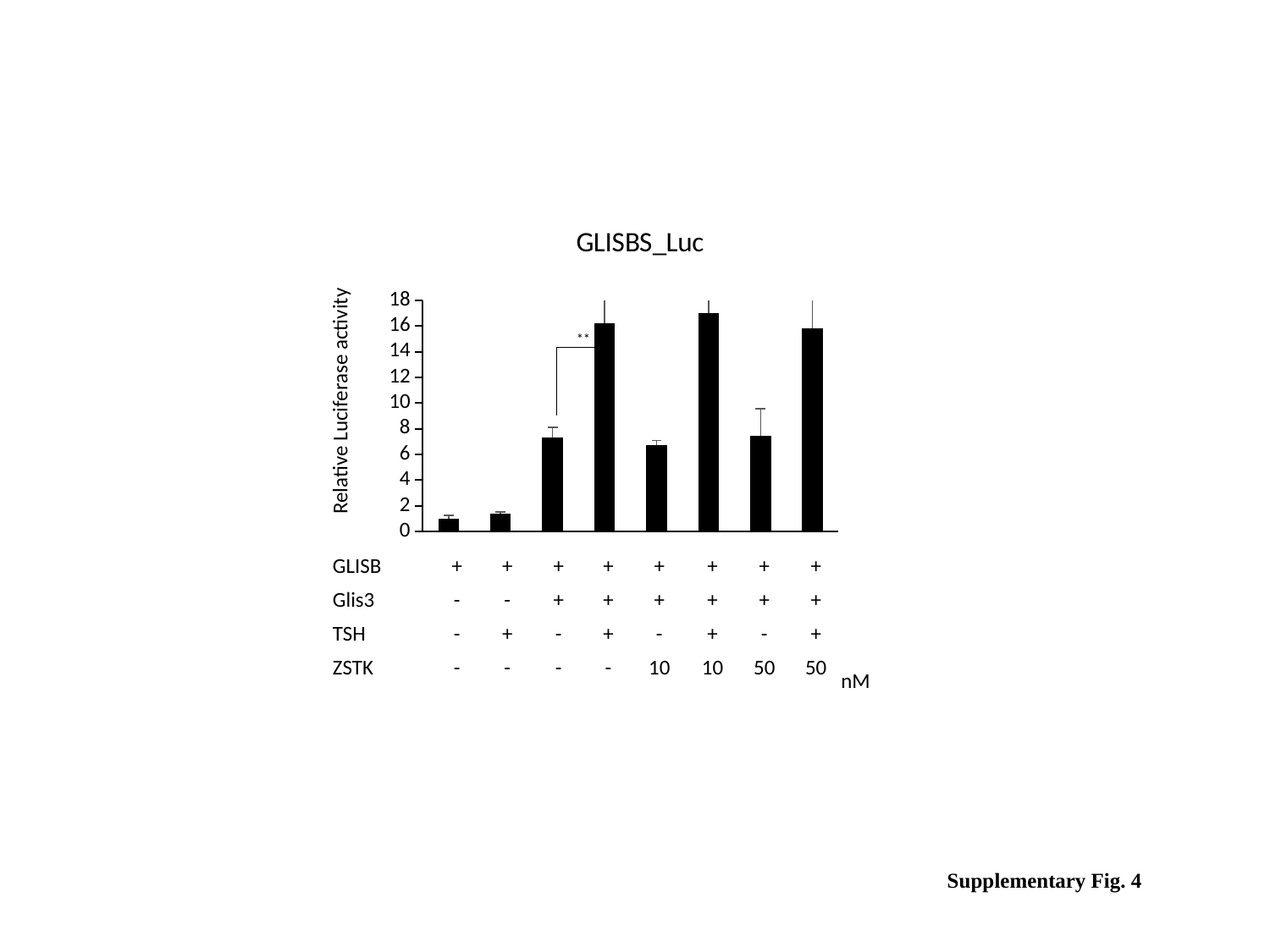

### Chart: GLISBS_Luc
| Category | |
|---|---|
| S1 | 1.0 |
| S2 | 1.402639302541494 |
| S3 | 7.299423736813982 |
| S4 | 16.199684433052223 |
| S5 | 6.71543544611915 |
| S6 | 17.0346487499848 |
| S7 | 7.454132592851075 |
| S8 | 15.7994998901002 |**
Relative Luciferase activity
| GLISB | + | + | + | + | + | + | + | + |
| --- | --- | --- | --- | --- | --- | --- | --- | --- |
| Glis3 | - | - | + | + | + | + | + | + |
| TSH | - | + | - | + | - | + | - | + |
| ZSTK | - | - | - | - | 10 | 10 | 50 | 50 |
nM
Supplementary Fig. 4

## Slide 5
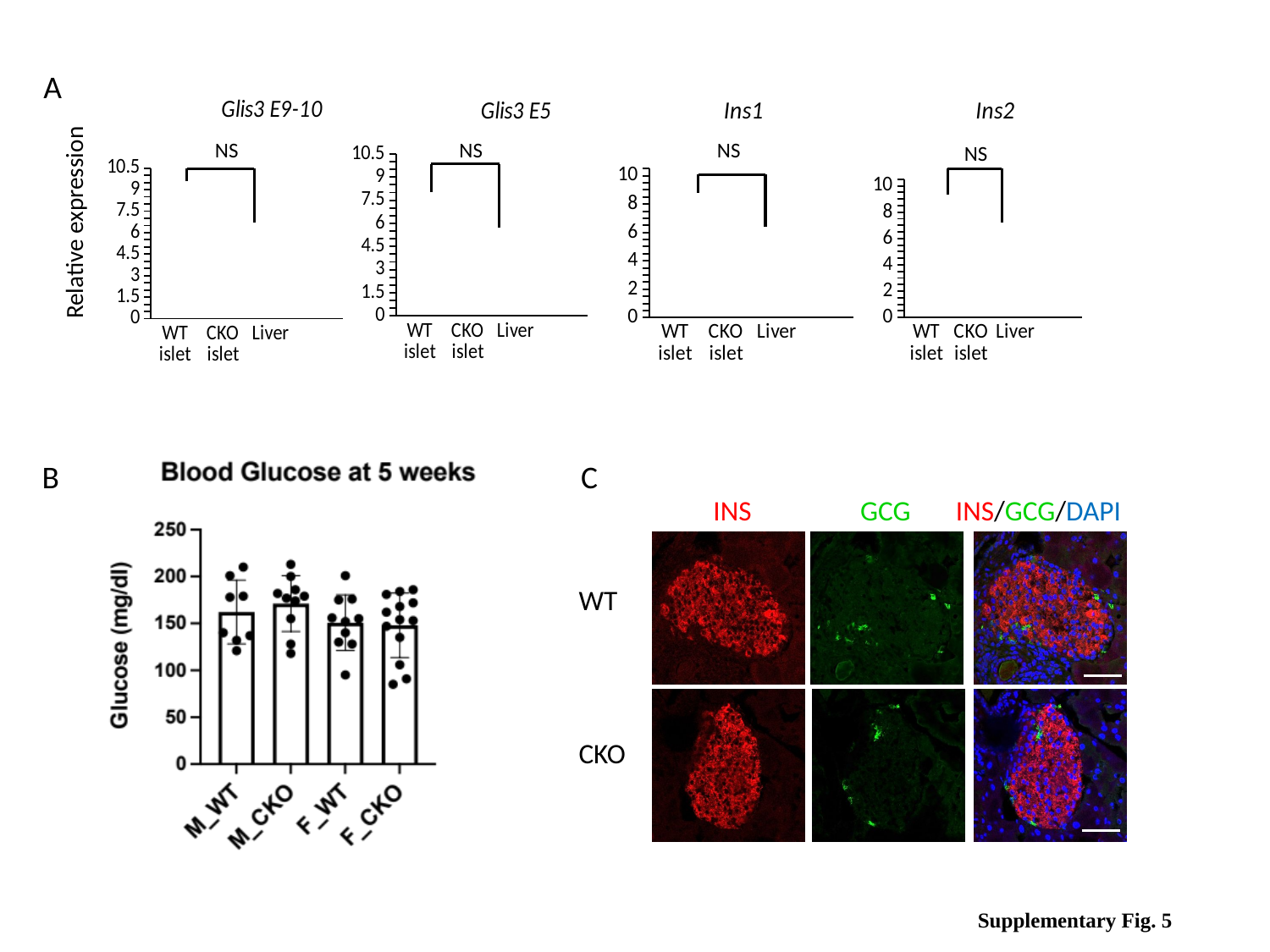

A
### Chart: Glis3 E9-10
| Category | AVE |
|---|---|
| WT islet | 1.0 |
| CKO islet | 0.5944222908121025 |
| Liver | 0.0415246786372222 |
### Chart: Glis3 E5
| Category | AVE |
|---|---|
| WT islet | 1.0 |
| CKO islet | 0.6925669877437791 |
| Liver | 0.023405502905257343 |
### Chart: Ins1
| Category | AVE |
|---|---|
| WT islet | 1.0 |
| CKO islet | 0.8222101783361742 |
| Liver | 5.089044928698372e-06 |
### Chart: Ins2
| Category | AVE |
|---|---|
| WT islet | 1.0 |
| CKO islet | 0.8239497713622363 |
| Liver | 9.024859875759659e-07 |NS
NS
NS
NS
Relative expression
B
C
INS
GCG
INS/GCG/DAPI
WT
CKO
Supplementary Fig. 5

## Slide 6
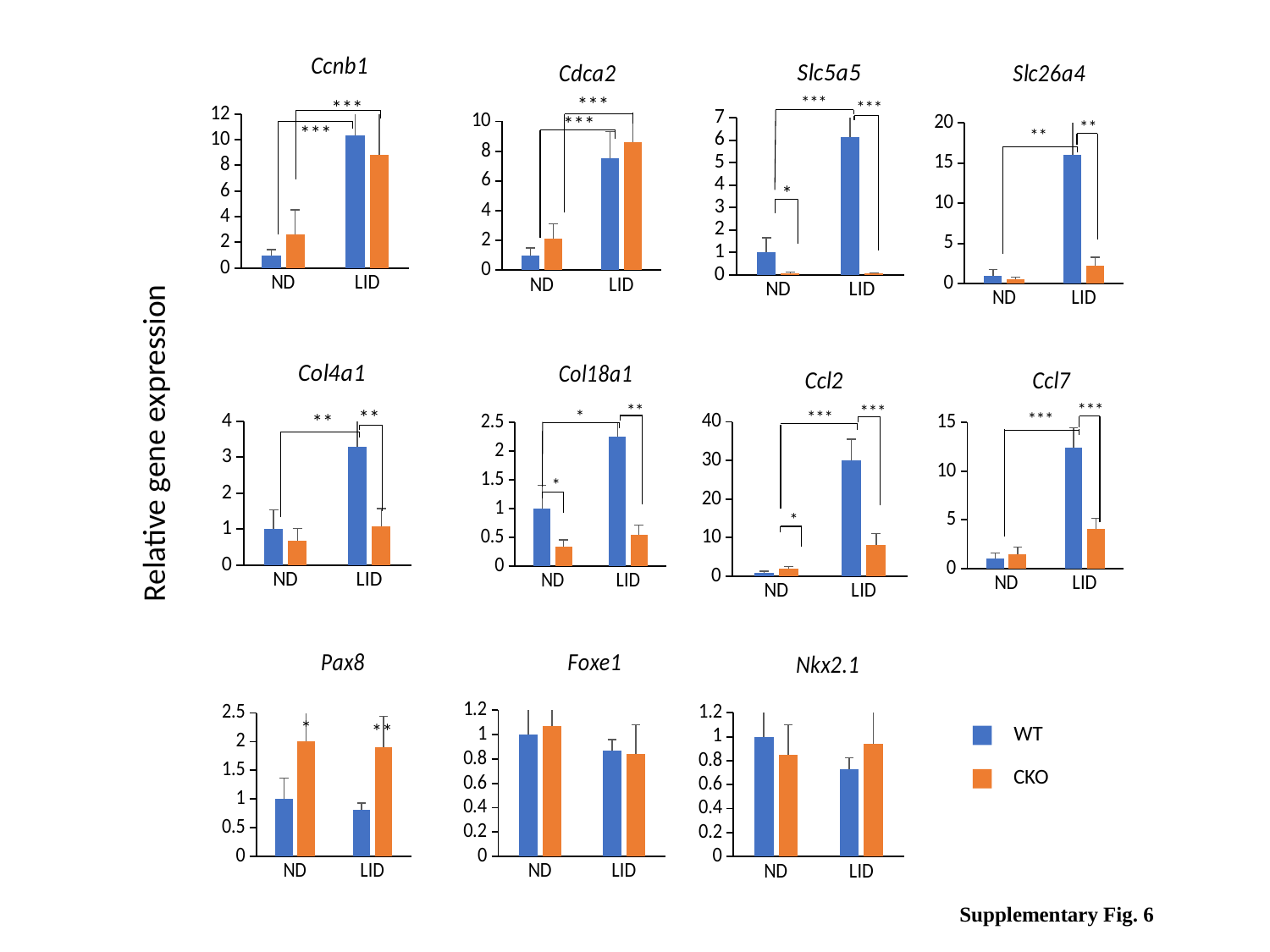

### Chart: Ccnb1
| Category | WT | CKO |
|---|---|---|
| ND | 1.0 | 2.586004724989272 |
| LID | 10.32738157421485 | 8.829315963682445 |
### Chart: Cdca2
| Category | WT | CKO |
|---|---|---|
| ND | 1.0 | 2.14844545095941 |
| LID | 7.549177522122046 | 8.60872080813835 |
### Chart: Slc5a5
| Category | WT | CKO |
|---|---|---|
| ND | 1.0 | 0.06887586105493229 |
| LID | 6.142839879330097 | 0.0679477292193442 |
### Chart: Slc26a4
| Category | WT | CKO |
|---|---|---|
| ND | 1.0 | 0.5503874806657968 |
| LID | 16.014169672841003 | 2.2538914251828643 |***
***
***
***
***
**
***
**
*
### Chart: Col4a1
| Category | WT | CKO |
|---|---|---|
| ND | 1.0 | 0.6882563233655316 |
| LID | 3.295505343078816 | 1.0818334284609357 |
### Chart: Col18a1
| Category | WT | CKO |
|---|---|---|
| ND | 1.0 | 0.3348838610143314 |
| LID | 2.2541810620637914 | 0.5444963311347379 |
### Chart: Ccl2
| Category | WT | CKO |
|---|---|---|
| ND | 1.0 | 1.9735589385886008 |
| LID | 29.92592545406375 | 8.227354611387973 |
### Chart: Ccl7
| Category | WT | CKO |
|---|---|---|
| ND | 1.0 | 1.4517908699010376 |
| LID | 12.439072986416466 | 4.114120374201231 |***
**
*
*
***
**
**
***
***
Relative gene expression
*
### Chart: Pax8
| Category | WT | CKO |
|---|---|---|
| ND | 1.0 | 2.008566531607901 |
| LID | 0.8109269116539017 | 1.9039641235503517 |
### Chart: Foxe1
| Category | WT | CKO |
|---|---|---|
| ND | 1.0 | 1.070941254902185 |
| LID | 0.8698937000836132 | 0.8414612111271833 |
### Chart: Nkx2.1
| Category | WT | CKO |
|---|---|---|
| ND | 1.0 | 0.8479784520097375 |
| LID | 0.730837096171855 | 0.9403454405546772 |*
**
WT
CKO
Supplementary Fig. 6

## Slide 7
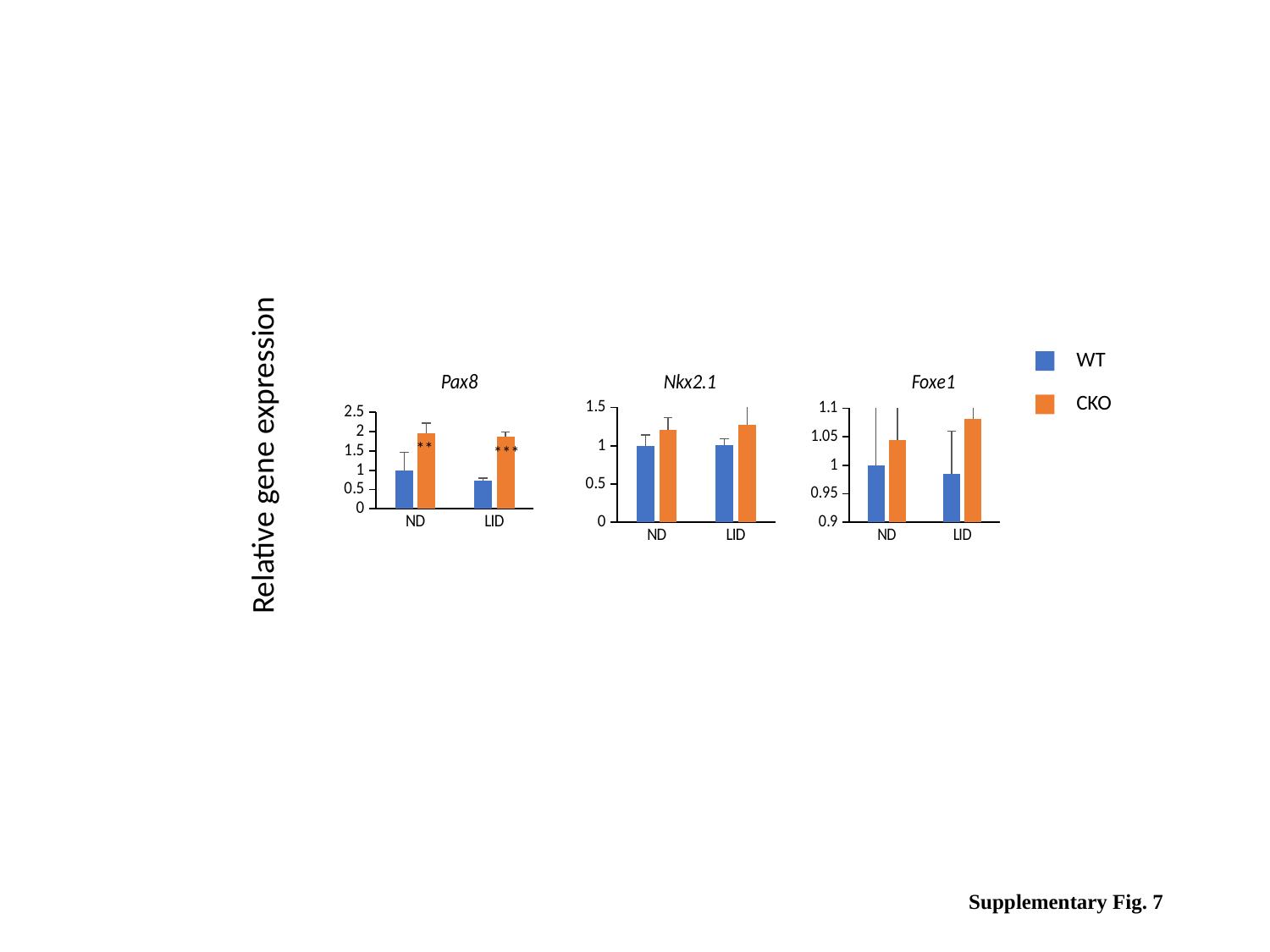

WT
CKO
### Chart: Pax8
| Category | WT | CKO |
|---|---|---|
| ND | 1.0 | 1.9635896452386037 |
| LID | 0.7320869170527958 | 1.8754418572275628 |
### Chart: Nkx2.1
| Category | WT | CKO |
|---|---|---|
| ND | 1.0 | 1.2109765307388192 |
| LID | 1.0034531670466955 | 1.2754651678368154 |
### Chart: Foxe1
| Category | WT | CKO |
|---|---|---|
| ND | 1.0 | 1.0436170533455251 |
| LID | 0.9847596970885818 | 1.0806173114416109 |Relative gene expression
**
***
Supplementary Fig. 7
